# Persistent *Burkholderia cepacia* Bacteremia in Reconstructive Surgery: Resistance to Ceftazidime/Avibactam and Co‐Trimoxazole With Risk of Infective Endocarditis—A Case Report

**DOI:** 10.1002/ccr3.71732

**Published:** 2025-12-22

**Authors:** Chukwuka Elendu, Dependable C. Amaechi, Tochi C. Elendu, Emmanuel C. Amaechi, Ijeoma D. Elendu, Adaobi S. Ikedilo

**Affiliations:** ^1^ Federal University Teaching Hospital Owerri Nigeria; ^2^ Igbinedion University Okada Nigeria; ^3^ Imo State University Owerri Nigeria; ^4^ Madonna University Elele Nigeria; ^5^ American University of Antigua St. John's Antigua and Barbuda

**Keywords:** bacterial multidrug resistance, *Burkholderia cepacia*, sepsis, skin grafting, wound infection

## Abstract

Persistent *Burkholderia cepacia* bacteremia in soft tissue infections creates diagnostic and therapeutic challenges due to its resistance profile and potential for endovascular involvement. Early culture‐guided therapy, multidisciplinary coordination, and vigilance for complications, such as infective endocarditis, are essential for achieving favorable outcomes in reconstructive surgical settings.

## 1 Introduction and Background

*Burkholderia cepacia* complex (Bcc) is an opportunistic group of Gram‐negative, non‐fermenting bacilli that pose significant therapeutic challenges, particularly in immunocompromised patients and individuals with underlying structural defects or indwelling devices [1]. Although it is most commonly associated with pulmonary infections in cystic fibrosis patients, Bcc has emerged as a cause of serious bloodstream infections, particularly in hospital settings, due to its intrinsic resistance to multiple antibiotics and its ability to survive in disinfectants and aqueous environments [2, 3].

Bcc bacteremia is associated with high morbidity, prolonged hospital stays, and substantial therapeutic dilemmas, primarily when resistance extends to last‐resort agents such as ceftazidime/avibactam and co‐trimoxazole [4]. These agents are often used as part of combination therapy against multidrug‐resistant Gram‐negative organisms, and resistance to both severely limits therapeutic options [5]. Furthermore, persistent bacteremia increases the risk of secondary complications such as infective endocarditis, particularly in surgical patients with prosthetic materials or recent tissue manipulation [6].

Our report describes a rare and clinically significant case of persistent *Burkholderia cepacia* bacteremia following reconstructive surgery, characterized by resistance to ceftazidime/avibactam and co‐trimoxazole and complicated by suspected infective endocarditis. The case highlights the diagnostic and therapeutic challenges in managing Bcc infections, underscoring the importance of antimicrobial stewardship and timely infectious disease consultation in such scenarios.

## 2 Case Presentation

The patient, a 35‐year‐old male farmer with no significant underlying comorbidities apart from a remote history of appendectomy, initially presented to our outpatient department with a constellation of symptoms that had evolved over several weeks. He reported a four‐week history of a non‐healing ulcer on the left leg, which had developed following minor trauma sustained while working on his farm. The wound had gradually worsened despite empirical outpatient treatment with oral amoxicillin‐clavulanate followed by metronidazole. Five days before admission, he began experiencing high‐grade fevers, malaise, intermittent cough, mild shortness of breath, and increasing pain in the affected leg. Given the progressive nature of these symptoms, he was referred for inpatient management.

On admission, he was febrile with a body temperature of 38.2°C, tachycardic with a pulse of 112 beats per minute, and hypotensive with a blood pressure of 90/60 mmHg. Clinical examination revealed an offensive, slough‐covered ulcer on the anterior aspect of the left leg, characterized by unhealthy granulation tissue and evidence of peri‐wound inflammation. Auscultation of the chest revealed coarse breath sounds bilaterally, although no focal crepitations or signs of consolidation were evident. A chest X‐ray later confirmed the presence of bilateral patchy infiltrates suggestive of either early pneumonia or an inflammatory response to sepsis. Limb radiographs showed no evidence of osteomyelitis or foreign bodies.

Initial laboratory investigations demonstrated anemia, leukocytosis with neutrophilia, and mildly elevated liver transaminases. In light of his septic presentation and the risk of polymicrobial wound infection, empiric broad‐spectrum antibiotic therapy was initiated, comprising intravenous ceftazidime/avibactam, clindamycin, and metronidazole. Fluid resuscitation, supportive care, and nutritional support were also provided. Blood cultures and wound swabs were promptly collected for microbiological analysis.

**FIGURE 1:**
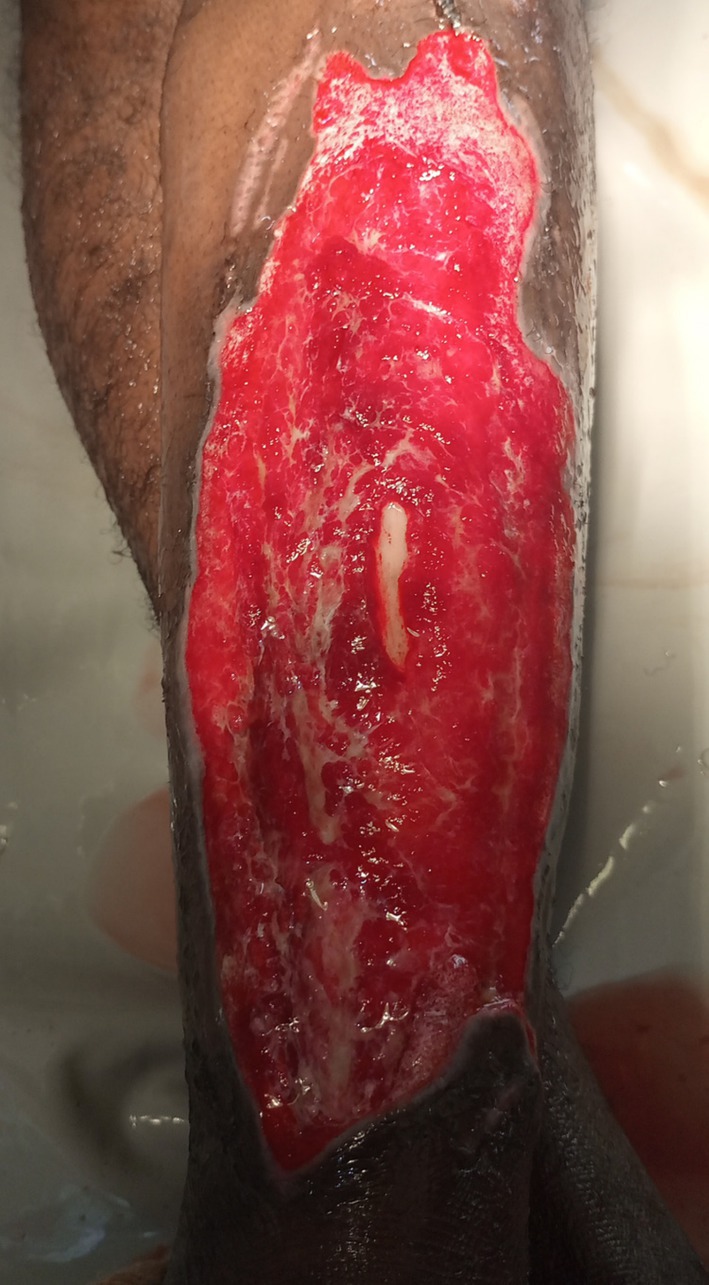
Chronic leg ulcer before surgical intervention, highlighting the extent of tissue damage and surrounding inflammation.

**FIGURE 2:**
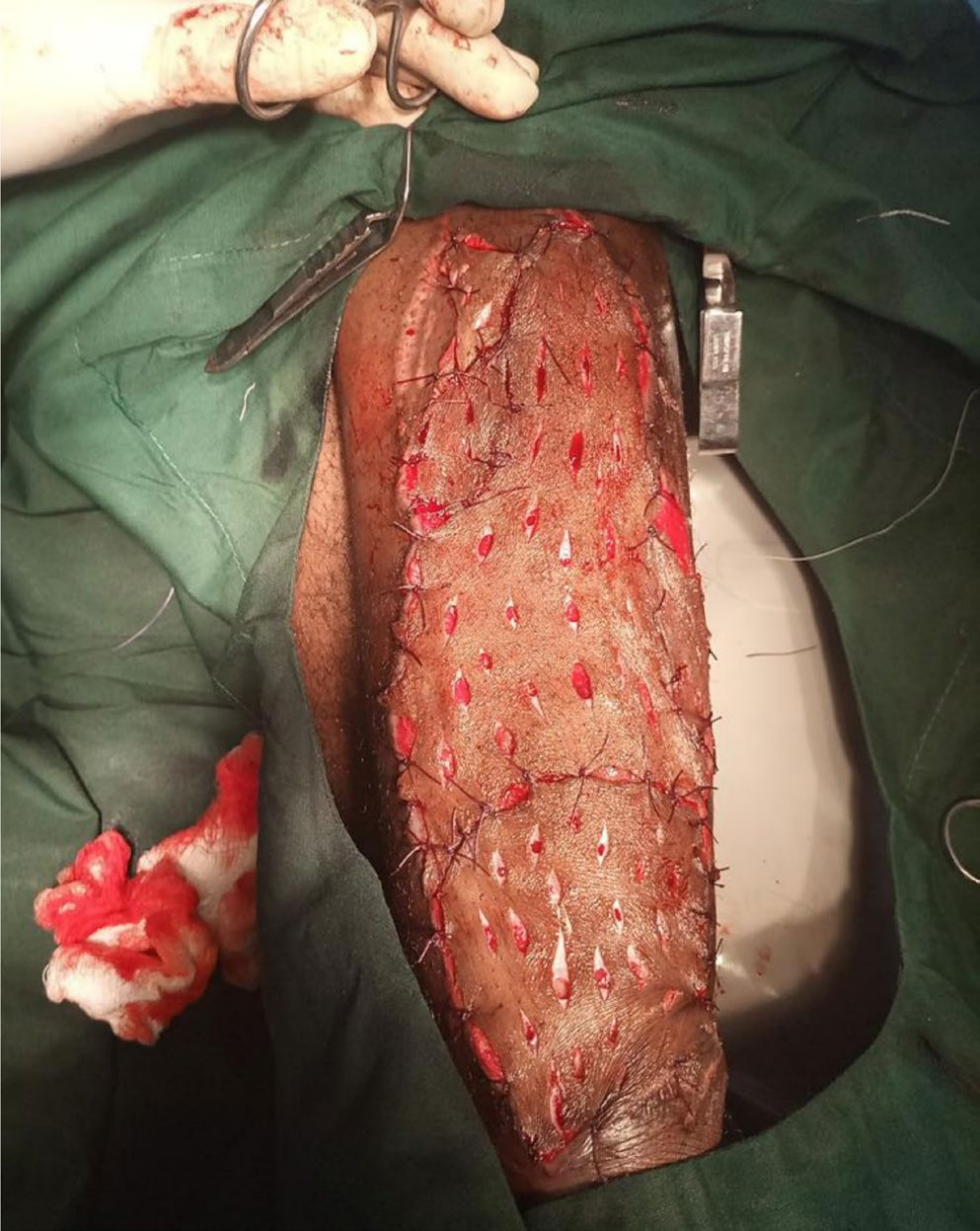
Autogenous split‐thickness skin grafting procedure performed on the chronic leg ulcer.

**FIGURE 3:**
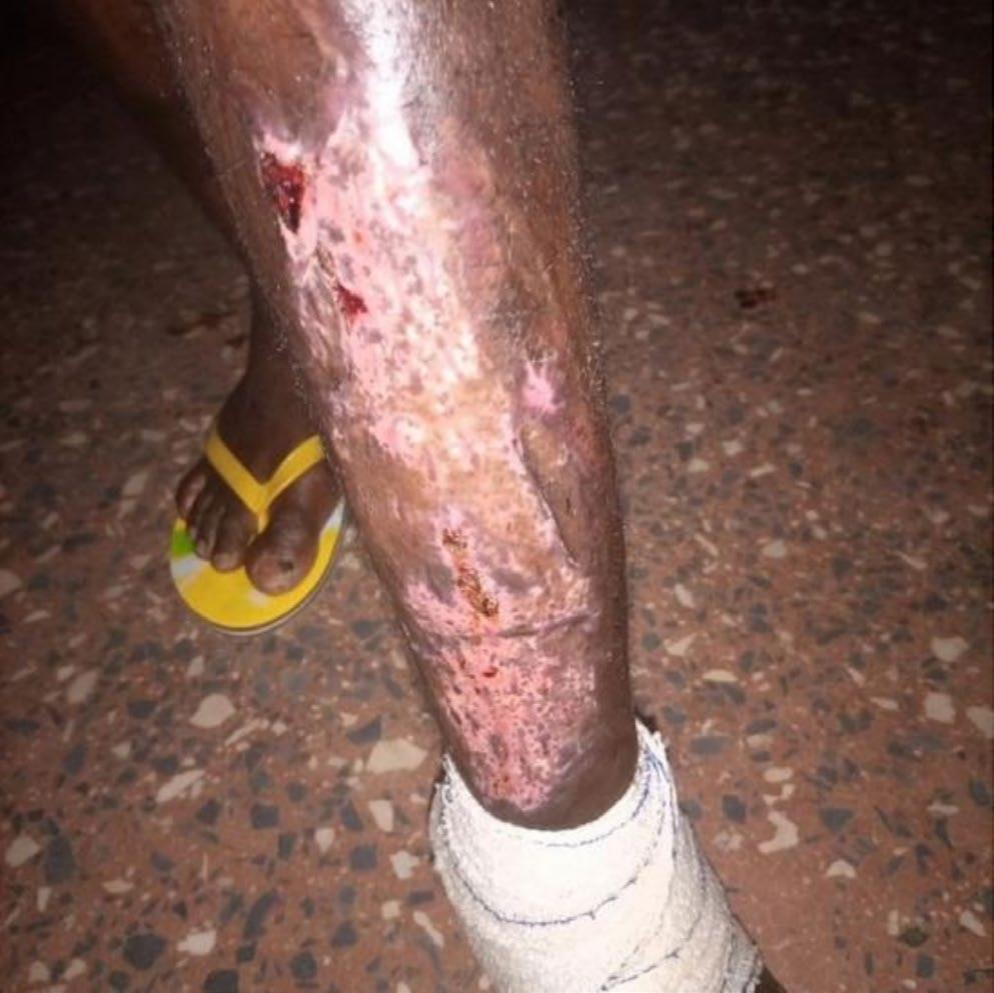
Postoperative appearance of the skin graft demonstrating early integration with surrounding tissue.

By the fifth hospital day, he had shown partial clinical improvement—blood pressure had normalized, cough and breathlessness were resolving—but he remained febrile, lethargic, and mildly dyspneic. With no culture results available yet, the surgical team proceeded with bedside irrigation of the ulcer using copious amounts of warm saline. The wound was then debrided thoroughly under sterile conditions (Figure 1). On the following day, once the wound bed appeared adequately prepared, the patient underwent a split‐thickness skin grafting procedure using an autogenous graft harvested from the opposite thigh (Figure 2). The graft appeared well‐integrated during the early postoperative period (Figure 3). Wound inspections over the next few days revealed progressive adherence, with mild serous drainage but no overt signs of graft rejection.

**FIGURE 4:**
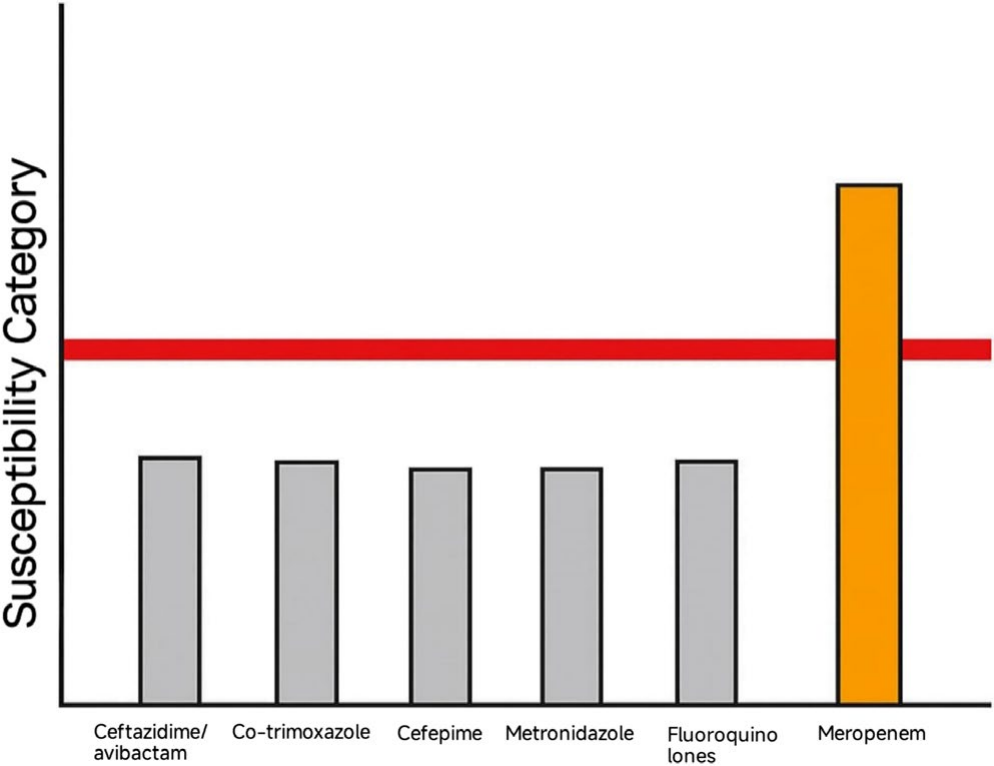
Antibiotic susceptibility profile of the *Burkholderia cepacia* complex isolate showing resistance and intermediate susceptibility to commonly used antimicrobials.

Despite this, the patient's fever persisted. On postoperative day 5 (hospital day 12), he remained febrile at 38.9°C and clinically fatigued. The delayed blood culture results became available, identifying Bcc as the causative agent. The isolate exhibited resistance to multiple agents, including ceftazidime/avibactam, co‐trimoxazole, cefepime, metronidazole, and fluoroquinolones, with only intermediate susceptibility to meropenem. This resistance profile, illustrated in Figure 4, matched the wound swab culture results, confirming the wound as the likely source of bacteremia.

**FIGURE 5:**
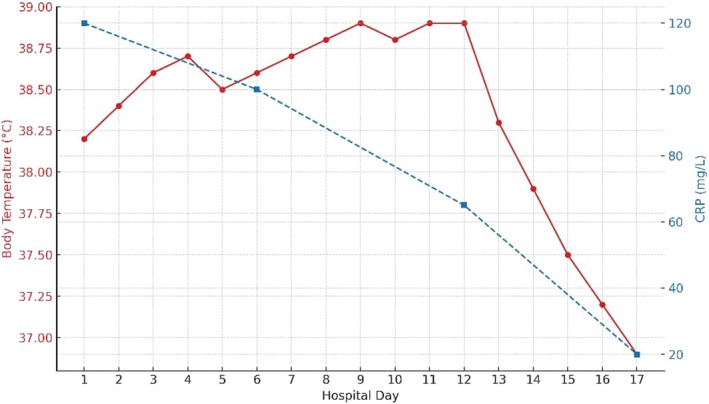
Daily temperature and CRP trends throughout the patient's hospitalization.

Additionally, Actinomyces species were isolated from the wound swab, demonstrating sensitivity only to carbapenems, further complicating the antimicrobial selection. Given the persistent bacteremia and the patient's prolonged fever despite therapy, clinical suspicion for secondary complications, including infective endocarditis, was raised. However, transthoracic echocardiography was performed and showed no definitive vegetations. The presence of *Burkholderia cepacia* bacteremia—a known endovascular pathogen—necessitated close cardiac monitoring and ongoing evaluation for endocardial involvement. Serial clinical reviews and laboratory markers later suggested resolution of systemic infection, and given the absence of new cardiac murmurs or echocardiographic changes, infective endocarditis was ultimately considered unlikely. This clinical trajectory is illustrated in Figure 5, which displays the patient's daily body temperature and CRP levels throughout hospitalization; however, the initial concern underscored the importance of vigilance in patients with persistent *Burkholderia cepacia* bacteremia.

In response, the empiric antibiotic regimen was discontinued, and the patient was transferred to a high‐dependency unit for close monitoring and targeted therapy. He was started on intravenous meropenem at 1 g every 8 h. This change aligned with the available susceptibility profile and contemporary guidance established by the Infectious Diseases Society of America (IDSA) for the management of multidrug‐resistant Gram‐negative infections, although the intermediate susceptibility required cautious optimism and close surveillance [1].

Over the next 5 days, the patient showed substantial improvement. He became afebrile, regained appetite, and his energy levels returned to baseline. Wound inspection showed continued graft viability with clean margins and no evidence of superimposed infection. Repeat blood cultures obtained after 5 days of meropenem therapy showed no growth. This sterile culture result, alongside sustained clinical improvement, supported the effectiveness of meropenem as a salvage agent in this multidrug‐resistant setting.

**FIGURE 6:**
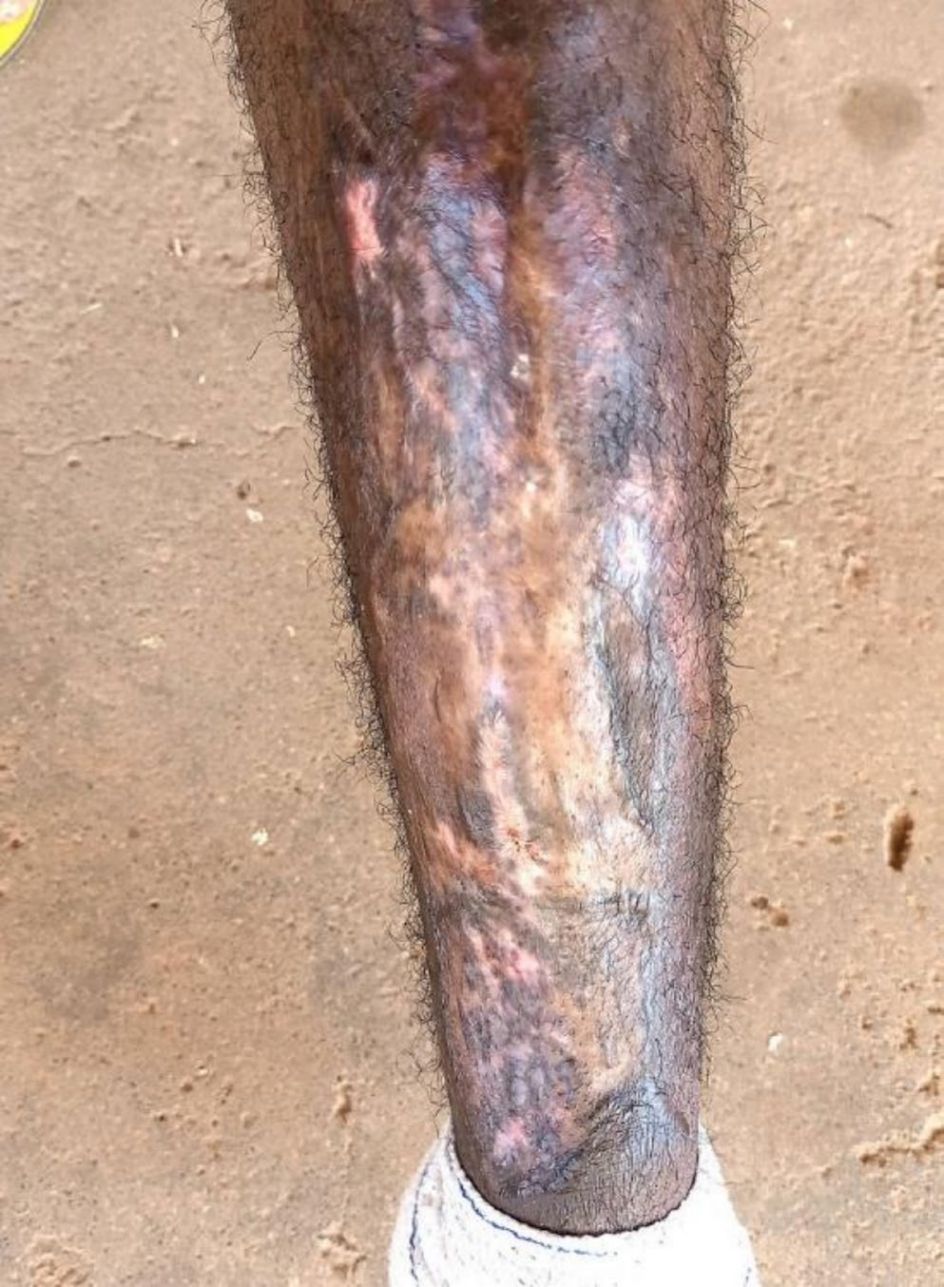
Healed skin graft site at 2‐week follow‐up, showing excellent cosmetic outcome and full graft integration without evidence of infection or rejection.

The patient was discharged home after a total of 17 days of inpatient care and instructed to continue a brief tapering course of oral supportive therapy. He was reviewed in the outpatient clinic 2 weeks post‐discharge. At follow‐up, the graft site had healed almost completely with excellent cosmetic and functional outcomes (Figure 6). There were no new complaints, and systemic examination was unremarkable. Repeat blood cultures remained negative, and his inflammatory markers had returned to normal levels.

The overall clinical course illustrates the diagnostic and therapeutic complexity posed by *Burkholderia cepacia* infections in the setting of reconstructive surgery (see Table 1). The case further underscores the importance of early culture acquisition, judicious use of antibiotics, and the integration of microbiological data into surgical decision‐making. As highlighted in this case, inappropriate outpatient antibiotic use may have contributed to the development of resistance, and empiric inpatient regimens failed to cover Bcc due to its unusual resistance profile adequately.

**TABLE 1:** Summary of clinical course, interventions, and microbiological findings.

Hospital day	Clinical status	Intervention	Microbiological results	Remarks
0 (Admission)	Fever, leg ulcer, cough, hypotension	Empiric IV antibiotics started (Ceftazidime/avibactam, clindamycin, metronidazole); blood/wound cultures sent	Pending	Suspected sepsis from soft tissue source
5	Partial improvement, still febrile	Wound debridement performed	Pending	Leg ulcer debrided at bedside
6	Post‐debridement	Split‐thickness skin grafting performed	Pending	Graft placed from opposite thigh
12	Persistent fever, bacteremia	Antibiotics switched to meropenem; moved to high‐dependency care	*B. cepacia* isolated (MDR); Actinomyces isolated from wound swab	Suspected endovascular involvement
17	Afebrile, stable, improving graft	Discharged with outpatient follow‐up plan	Blood cultures negative after meropenem	Clinical recovery
Follow‐up (2 weeks)	Fully recovered	Continued wound care and monitoring	Repeat cultures negative	Graft healed; no recurrence

*Note:* Source: Authors' creations.

Abbreviations: *B. cepacia*, *Burkholderia cepacia*; MDR, multidrug‐resistant.

Critically, this case highlights the importance of current antimicrobial stewardship principles and existing infectious disease guidelines, particularly those from the IDSA, which recommend prompt identification and de‐escalation based on culture results in cases of Gram‐negative bacteremia [1]. The patient's recovery without surgical re‐intervention and the resolution of systemic infection reinforce the importance of early identification and targeted therapy, even in settings with limited access to advanced antibiotics.

All the initial clinical assessments and documentation were handled by the corresponding author. He coordinated care across surgical and infectious disease teams, providing detailed documentation of the patient's history, antimicrobial exposures, and the timing of surgical interventions. This documentation significantly contributed to establishing the causative sequence of events and guiding appropriate management.

## 3 Differential Diagnosis, Treatment Plan, and Follow‐Up

The patient's initial presentation with prolonged fever, cough, lethargy, and a chronic leg ulcer following trauma raised immediate concerns for systemic infection with a likely wound source. The constellation of findings—including fever, tachycardia, hypotension, elevated white blood cell count, and imaging showing bilateral patchy infiltrates—necessitated a broad differential diagnosis (see Table 2) [6]. Septicemia originating from a soft tissue infection was high on the list, with the non‐healing ulcer acting as a clear nidus. However, given his respiratory symptoms and radiographic findings, community‐acquired pneumonia or sepsis with pulmonary involvement also required consideration [7, 8]. Atypical bacterial infections and tuberculosis were part of the initial respiratory differential but were considered less likely due to the acute evolution of symptoms [9]. Although no signs of osteomyelitis were evident on limb radiography, the duration of the wound and its malodorous nature meant that deep‐seated infection could not be ruled out at the outset.

**TABLE 2:** Differential diagnosis considered on admission.

Condition	Supporting features	Reasons for exclusion/deprioritization
Soft tissue sepsis	Chronic leg ulcer, fever, leukocytosis, hypotension	Confirmed by wound swab and blood cultures
Community‐acquired pneumonia	Cough, breathlessness, bilateral patchy infiltrates on CXR	No focal crepitations, rapid resolution after source control, negative sputum cultures
Tuberculosis	Chronic cough, endemic setting	No weight loss or night sweats; acute course; negative AFB and GeneXpert (if tested)
Necrotizing fasciitis	Ulcer with slough and systemic toxicity	No crepitus, no rapidly spreading infection, pain not disproportionate
Osteomyelitis	Chronic ulcer overlying tibia	Radiographs showed no bony involvement
Infective endocarditis	Persistent fever and bacteremia; *B. cepacia* is endovascular pathogen	No murmurs; negative TTE; resolution with antibiotics without signs of embolic events

*Note:* Source: Authors' creations.

Abbreviations: *B. cepacia*, *Burkholderia cepacia*; CXR, chest X‐ray; TTE, transthoracic echocardiogram.

There was also concern for necrotizing fasciitis, given the appearance of the ulcer and systemic toxicity; however, the absence of crepitus, rapid progression, or severe pain disproportionate to findings helped to de‐prioritize this possibility [10]. In the background of prolonged fever and evolving sepsis, the team also considered endovascular sources of infection, including infective endocarditis, particularly after initial blood cultures remained pending and the patient failed to respond adequately to therapy. Although clinical examination revealed no new murmurs or peripheral stigmata of endocarditis, the risk remained elevated due to persistent bacteremia. A transthoracic echocardiogram was obtained, which showed no vegetations or structural abnormalities, but *Burkholderia cepacia* is known for its endovascular tropism, warranting ongoing vigilance.

Once microbiological confirmation was obtained, the patient's management plan was adjusted based on the susceptibility profile and contemporary guidelines. Close monitoring continued, with attention to signs of endovascular involvement and graft viability. Fever resolved, systemic parameters improved, and serial blood cultures showed clearance of bacteremia, confirming microbiological and clinical resolution. Graft inspections demonstrated steady healing without signs of superimposed infection.

The total inpatient stay lasted 17 days, during which the patient transitioned from systemic sepsis to complete recovery. He was discharged with instructions for wound protection, hygiene, and elevation to support graft healing. Counseling was provided to discourage the use of antibiotics without supervision, which may have contributed to the observed resistance pattern. Follow‐up at 2 weeks revealed that he remained afebrile, the graft had integrated well, and there were no new complaints or signs of infection. Inflammatory markers normalized, and repeat cultures remained negative.

This case illustrated the complexity of managing persistent bacteremia in the setting of soft tissue infection, particularly when the pathogen exhibits an unusual resistance profile. The initial differential diagnosis was broad, and a stepwise narrowing of possibilities—guided by evolving clinical findings and diagnostic results—was critical to reaching the final diagnosis. The suspicion for infective endocarditis, although not confirmed, reflected prudent clinical vigilance given the known characteristics of *B. cepacia* bacteremia. The multidisciplinary approach, combining timely surgical intervention, culture‐directed therapy, and close follow‐up, contributed to the favorable outcome.

By the end of follow‐up, the patient had regained full use of his limb without signs of recurrence, endocarditis, or chronic complications such as osteomyelitis. He resumed his daily activities, including farming, and continued to receive support through outpatient services. This case highlights the importance of timely diagnosis, personalized management, and effective stewardship in the treatment of rare but clinically significant pathogens in reconstructive surgery.

## 4 Discussion

Persistent Bcc bacteremia presents significant clinical challenges, particularly during the postoperative period of reconstructive surgery. This case illustrates the complex interplay between chronic wound management, antibiotic resistance, and vigilance for potential complications such as infective endocarditis. *Burkholderia cepacia* is a Gram‐negative, non‐fermenting bacillus, notorious for its resistance to multiple classes of antibiotics and its ability to survive in moist environments, making it a formidable nosocomial pathogen [1, 2]. While traditionally associated with pulmonary infections in patients with cystic fibrosis, its role in bacteremia and endovascular infections, particularly among hospitalized and immunocompromised patients, has been increasingly recognized [3].

In this patient, the chronicity of the leg ulcer and a background of empirical, possibly suboptimal antibiotic use before presentation created a fertile ground for colonization by resistant organisms. Chronic wounds, particularly those exposed to environmental pathogens or treated empirically in community settings, have been documented as significant portals for systemic infections, including bacteremia [4, 11]. The wound's failure to heal despite initial outpatient therapy and its subsequent colonization by multidrug‐resistant Bcc reflect this risk. The intraoperative findings and the need for autogenous split‐thickness skin grafting further underscore the complexity of the wound bed and the surgical team's efforts to enhance tissue regeneration through the application of vascularized grafts [5]. However, post‐grafting bacteremia raised immediate concerns about graft viability and the possibility of deeper‐seated or disseminated infection.

The organism's resistance profile played a pivotal role in shaping the therapeutic course. Resistance to ceftazidime/avibactam and co‐trimoxazole—a combination often used against Gram‐negative organisms—complicated initial management. In vitro susceptibility to meropenem enabled rational antibiotic escalation, albeit with intermediate susceptibility, necessitating close clinical monitoring. *B. cepacia* 's intrinsic and acquired resistance mechanisms, including efflux pumps and β‐lactamases, limit the utility of many conventional antimicrobials, leaving clinicians with fewer therapeutic options [2, 12]. Although minocycline and other agents have been suggested in some studies as alternatives, their clinical efficacy in deep tissue and endovascular infections remains under‐researched, limiting their use in high‐risk scenarios such as bacteremia or post‐surgical infection [6].

Beyond antimicrobial resistance, *B. cepacia* 's ability to form biofilms significantly hinders treatment. Biofilm formation on wound surfaces and possibly on graft material reduces antibiotic penetration and promotes persistence, contributing to delayed healing and recurrent bacteremia [7]. In this case, despite prompt surgical debridement and grafting, the infection persisted, requiring further vigilance. Although no additional surgical intervention was needed after meropenem, close monitoring of wound integrity and systemic markers was necessary. The eventual clinical resolution, evidenced by normalization of inflammatory markers, negative blood cultures, and successful graft healing, highlights the effectiveness of coordinated care.

This case also highlights the important, yet often underappreciated, risk of endovascular involvement in *B. cepacia* bacteremia. Although transthoracic echocardiography was negative for vegetations and no stigmata of endocarditis were present on physical examination, clinical suspicion for infective endocarditis remained justified. Persistent bacteremia, the known endovascular affinity of *B. cepacia* , and the delayed resolution of fever necessitated careful cardiovascular evaluation [12]. While a negative echocardiogram reduced the likelihood of infective endocarditis, it did not entirely exclude it, especially considering the limitations of transthoracic imaging in detecting small vegetations. However, serial evaluations, absence of new murmurs, and progressive clinical improvement supported the conclusion that endocarditis did not occur in this patient.

The diagnostic approach in this case benefited from the timely acquisition of blood and wound cultures, as well as the use of advanced microbiological methods. Matrix‐assisted laser desorption/ionization–time‐of‐flight mass spectrometry (MALDI‐TOF MS), increasingly available in modern microbiology laboratories, enables the rapid and accurate identification of unusual pathogens, such as *B. cepacia* , facilitating early intervention [9]. Furthermore, targeted therapy based on susceptibility profiles enabled clinical recovery, reinforcing the principle that empiric antibiotic therapy should constantly be reassessed once culture results are available—a key recommendation in infectious disease stewardship frameworks, including those outlined by the IDSA [10].

This case reinforces several core clinical lessons. First, in chronic wounds that are unresponsive to empirical therapy, early microbiological evaluation is essential to prevent a delayed diagnosis of resistant organisms. Second, suspicion for endovascular involvement should be maintained in all patients with persistent Gram‐negative bacteremia, even in the absence of overt signs of endocarditis. Third, antimicrobial resistance in *B. cepacia* requires that clinicians remain up to date on evolving susceptibility trends and emerging therapies, as resistance to commonly used agents like co‐trimoxazole and ceftazidime/avibactam is increasingly documented [1, 12]. Fourth, multidisciplinary collaboration—among surgeons, infectious disease specialists, cardiologists, and microbiologists—is crucial for favorable outcomes in such complex cases. In our case, careful coordination between the surgical team and infection specialists ensured timely surgical debridement, appropriate antibiotic selection, and follow‐up surveillance.

While this case resulted in favorable outcomes, it raises broader questions about the management of multidrug‐resistant organisms in low‐resource settings. Access to advanced diagnostics, newer antibiotics, and trained personnel in infectious diseases is often limited in such environments. Moreover, the over‐the‐counter availability of antibiotics and empirical prescribing practices may contribute to the escalation of resistance trends [13, 14]. Public health strategies aimed at curbing antimicrobial misuse, strengthening diagnostic capacity, and reinforcing infection prevention and control measures are essential to limiting the spread of resistant pathogens, such as *B. cepacia* .

Finally, although our patient did not develop infective endocarditis, we do not claim that this case demonstrates the prevention of infective endocarditis. Instead, it underscores the importance of vigilance and the early adoption of risk mitigation strategies in the presence of pathogens known for endovascular adherence. Biofilm‐forming bacteria like *B. cepacia* are uniquely capable of seeding cardiac valves, particularly in hosts with underlying comorbidities or prosthetic materials [3, 7]. As such, timely intervention, guided by susceptibility testing and dynamic clinical assessment, likely mitigated—but did not eliminate—the risk.

## 5 Concluding Remarks

Managing *Burkholderia cepacia* bacteremia requires timely microbiological diagnosis, targeted antimicrobial therapy, and coordinated clinical care. Attention to potential complications such as endocarditis and prompt surgical intervention is essential to ensure recovery and reduce the risk of long‐term morbidity.

## Author Contributions

**Chukwuka Elendu:** conceptualization, investigation, project administration, supervision, validation, visualization, writing – original draft. **Dependable C. Amaechi:** data curation, methodology, writing – review and editing. **Tochi C. Elendu:** data curation, software, writing – review and editing. **Emmanuel C. Amaechi:** formal analysis, validation, writing – review and editing. **Ijeoma D. Elendu:** visualization, writing – review and editing. **Adaobi S. Ikedilo:** software, writing – review and editing.

## Funding

The authors have nothing to report.

## Disclosure

The views expressed in this report are solely those of the author(s) and do not represent the official positions of any affiliated institutions.

## Ethics Statement

The authors have nothing to report.

## Consent

The authors confirm that written informed consent was obtained from the patient for publication of this case report and accompanying clinical data.

## Conflicts of Interest

The authors declare no conflicts of interest.

## Data Availability

The authors have nothing to report.
