# Correction to “Persistent *Burkholderia cepacia* Bacteremia in Reconstructive Surgery: Resistance to Ceftazidime/Avibactam and Co‐Trimoxazole With Risk of Infective Endocarditis—A Case Report”

**DOI:** 10.1002/ccr3.72094

**Published:** 2026-02-15

Elendu C, Amaechi DC, Elendu TC, Amaechi EC, Elendu ID, Ikedilo AS. Persistent *Burkholderia cepacia* Bacteremia in Reconstructive Surgery: Resistance to Ceftazidime/Avibactam and Co‐Trimoxazole With Risk of Infective Endocarditis—A Case Report. *Clinical Case Reports*. 2025;14(1):e71732. https://doi.org/10.1002/ccr3.71732

In the originally published version of this article, the final paragraph of the Discussion section was incomplete. The paragraph originally read:

“Finally, although our patient did not develop infective endocarditis, we do not claim that this case demonstrates the prevention of infective endocarditis. Instead, it underscores the importance of vigilance and the early adoption of risk mitigation strategies in the presence of pathogens known for endovascular adherence. Biofilm‐forming bacteria like *B. cepacia* are uniquely capable of seeding cardiac valves, particularly in hosts with underlying comorbidities or prosthetic materials [3, 7]. As such, timely intervention, guided by susceptibility testing and dynamic clinical assessment, likely mitigated—but did not eliminate—the risk.”

The corrected paragraph should read:

“Finally, although our patient did not develop infective endocarditis, we do not claim that this case demonstrates the prevention of infective endocarditis. Instead, it underscores the importance of vigilance and the early adoption of risk mitigation strategies in the presence of pathogens known for endovascular adherence. Similar clinical presentations of *Burkholderia cepacia* bacteremia arising from chronic soft‐tissue wounds have been previously reported in the literature [15], reinforcing the relevance of this concern. Biofilm‐forming bacteria like *B. cepacia* are uniquely capable of seeding cardiac valves, particularly in hosts with underlying comorbidities or prosthetic materials [3, 7]. As such, timely intervention, guided by susceptibility testing and dynamic clinical assessment, likely mitigated—but did not eliminate—the risk.”

The reference cited as [15] in the corrected paragraph was missed in the original article and has now been included here.

These corrections do not affect the clinical interpretation or conclusions of the article. We apologize for this error.
